# Protecting children from rabies with education and pre-exposure prophylaxis: A school-based campaign in El Nido, Palawan, Philippines

**DOI:** 10.1371/journal.pone.0189596

**Published:** 2018-01-02

**Authors:** Raffy Deray, Cesar Rivera, Shiela Gripon, Corazon Ulanday, Maria Concepcion Roces, Anna Charinna Amparo, Michael Attlan, Clarisse Demont, Alexia Kieffer, Mary Elizabeth Miranda

**Affiliations:** 1 National Center for Disease Prevention and Control, Department of Health, Manila, Philippines; 2 Municipal Health Office, El Nido, Palawan, Philippines; 3 Department of Education, El Nido, Palawan, Philippines; 4 Global Alliance for Rabies Control, Laguna, Philippines; 5 Sanofi Pasteur, Lyon, France; Public Health England, UNITED KINGDOM

## Abstract

**Background:**

Rabies remains endemic in the Philippines. A study was conducted in El Nido, Palawan, Philippines to: (i) detect the true incidence of animal bites in school children aged 5–14 years using active surveillance and compare these data to estimates from the existing passive surveillance system, (ii) evaluate the impact of rabies prevention education and pre-exposure prophylaxis (PrEP) on animal bite incidence, and (iii) assess the health economic impact of the interventions.

**Methodology and principal findings:**

A cohort of 4,700 school children was followed-up for any suspect rabies exposures between January 2011 and December 2012. Data on animal bite incidence from the study cohort were compared to that obtained from a review of consultation records at the Animal Bite Treatment Center (ABTC). PrEP was offered to children in all 27 public elementary schools in El Nido (in January to February 2012). Teachers were given a manual for integrating rabies in the public elementary school curriculum during the school year 2012–13. Active surveillance of the cohort revealed a higher incidence of suspect rabies exposures than that from passive surveillance. Despite a decrease in the number of Category III bites, there was no significant decrease in overall bite incidence as a result of the interventions. However, there was an increase in rabies awareness among school children in all grade levels. There was also a high level of acceptability of PrEP. Children who received PrEP and subsequently were bitten only needed two booster doses for post-exposure prophylaxis, resulting in substantial cost-savings.

**Conclusions/significance:**

The true burden of animal bites remains underestimated in ABTC records. PrEP is advantageous in selected population groups, i.e. school-aged children in rabies endemic areas with limited access to animal and human rabies prevention services. Educating school children is beneficial. Strengthening veterinary interventions to target the disease at source is important.

## Introduction

It is widely recognized that the global numbers of human rabies deaths officially reported are greatly underestimated and reliable data indicating the true incidence of human rabies are scarce or non-existent in many countries [1–3].It is the poorest and often overlooked communities that are most at risk of exposure and death from rabies, and its control is often given a low priority by policymakers.

Dog rabies is endemic in the Philippines. The country ranks among the top ten countries worldwide for human rabies deaths. According to a report from the Department of Health National Rabies Prevention and Control Program (NRPCP) on human rabies and animal bite victims, in 2010, 257 rabies cases and 266,200 animal bites or suspect rabies exposures were recorded. A total of 365 animal bite treatment centers (ABTC) have been established by 2010 and strategically located nationwide to provide post exposure prophylaxis (PEP). The majority of potential rabies exposures resulted from dog bites and most exposures occurred in children < 15 years [4].

The Congress of the Philippines enacted a law, the Anti-Rabies Act of 2007, which declared the policy of the State to control, prevent the spread, and eventually eliminate human and animal rabies, and established the need for responsible pet ownership [5]. This law reinforced the National Rabies Prevention and Control Program that is implemented by a multi-sectoral committee chaired by the Department of Agriculture. The national program is a multi-agency effort in controlling and eliminating rabies in the country. Its component activities include: mass vaccination of dogs; establishment of a central database system for dog registration and vaccination; management of unregistered, stray and unvaccinated dogs; information and education campaigns on the prevention and control of rabies; provision of preventive rabies vaccination to high risk personnel and post-exposure prophylaxis to animal bite victims; provision of free routine immunization or pre-exposure prophylaxis (PrEP) to schoolchildren in areas where there is a high incidence of rabies; and promotion of responsible pet ownership. Section 4 of this law mandates the provision of free routine immunization or PrEP of schoolchildren aged 5 to 14 years in areas where there is high incidence of rabies and the integration of rabies prevention in the elementary school curriculum.

Anti-rabies vaccines can be given before (PrEP) or after (PEP) a rabies exposure. The guidelines for animal bite management [6] issued by the Philippines Department of Health in 2009 follows the recommendations released by the World Health Organization (WHO). WHO guidance is that people (especially children) travelling to or living in high risk areas should be offered PrEP. [7]

In the event of a bite, PEP is given on Days 0, 3, 7, 14 and 28 or 30 for the intramuscular (IM) regimen, or Days 0, 3, 7 and 28 or 30 for the intradermal (ID) regimen. PrEP is given on Days 0, 7 and 21 or 28 either IM or ID. PrEP provides economic benefits when targeted to high risk populations, since it reduces the PEP doses from five to two and it eliminates the need for Rabies Immune Globulin (RIG) [8].

**Fig 1:**
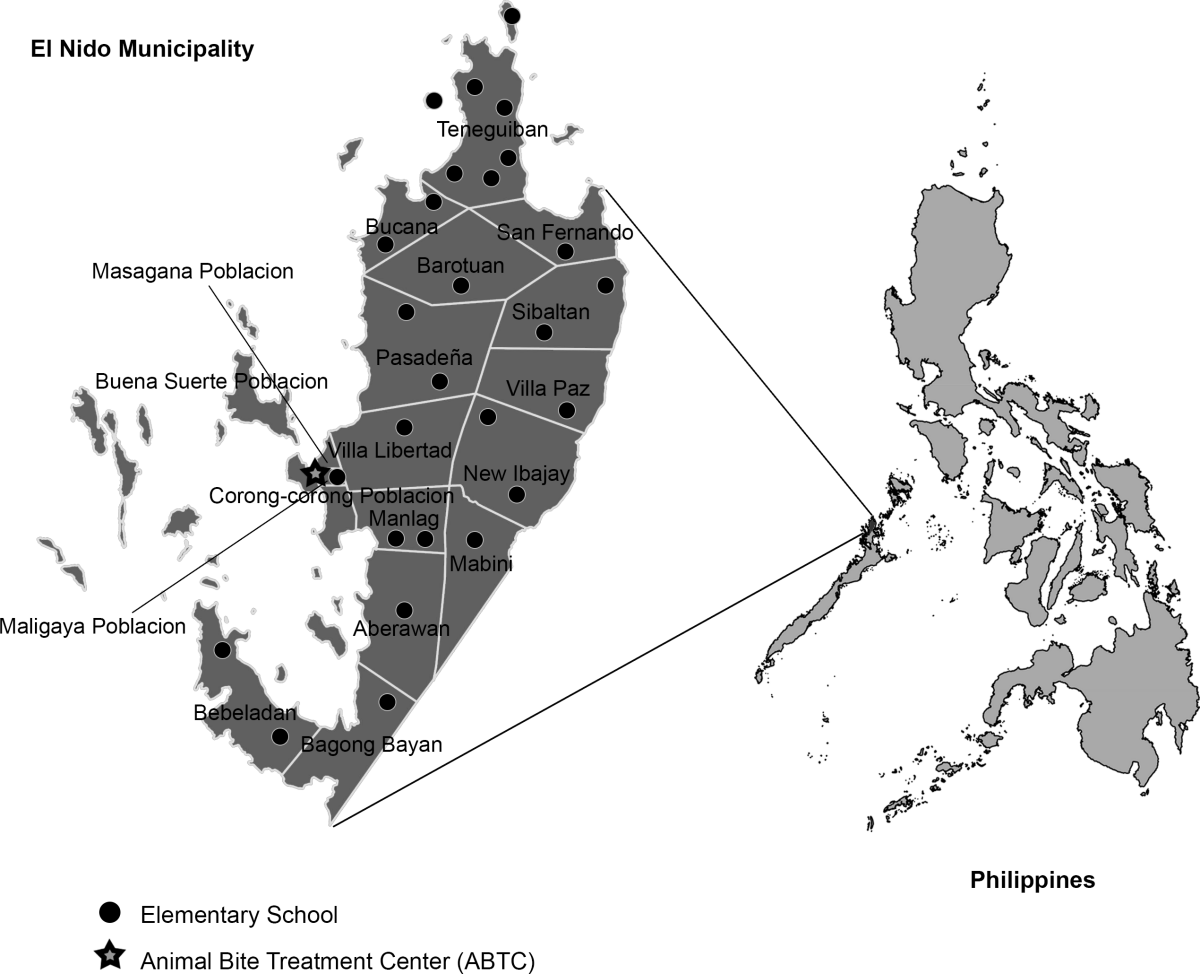
Map of El Nido, Palawan, Philippines showing villages and location of elementary schools and animal bite treatment center (ABTC).

The Municipality of El Nido was identified by the Department of Health NRPCP as the project site. It is a remote, rural area composed predominantly of agriculture and fishing communities in the northernmost part of the main island in the Province of Palawan (Fig 1). The estimated population in 2009 was 35,652 belonging to 6,807 households. El Nido has only one ABTC, located in the Municipal Health Office, which is the only source of PEP in the municipality. This center has a logbook of animal bite cases reported from the entire municipality, enabling follow-up of the study population. From 2005 to 2010, there were 826 consultations for animal bites at the ABTC, and 47% (389) of the patients were children < 15 years old. There were two reported human rabies deaths in 2009 in the municipality. Prior to the project initiation, no rabies education existed and there was very limited animal rabies control, thus children remained vulnerable to rabies, and the project was deemed beneficial to the community.

## Study objectives

In the frame of rabies PrEP intervention, this observational prospective study was conducted to evaluate the true incidence of animal bites in school children aged 5–14 years using active surveillance and compare this to estimates from the existing passive surveillance system. We also evaluated the impact of rabies PrEP interventions, i.e. disease prevention education and rabies PrEP immunization, on the incidence of animal bites, and consequently on the number and characteristics of the post-exposure prophylaxis and assessed the potential health economic impact of the interventions.

## Methods

The study encompassed a prospective observational assessment of the impact of preventive rabies vaccination and the implemention of a rabies prevention educational program in elementary school children in El Nido. The human rabies prevention program included rabies-specific teaching and provision of complete rabies PrEP vaccination. This was conducted in collaboration with the municipal and provincial local government units (LGU) of the project site, the Department of Health (DOH), Sanofi Pasteur and the Global Alliance for Rabies Control (GARC). DOH managed the admnistration of PrEP for the schoolchildren and the PEP for the animal bite patients, and ensured the continuous reporting as per the national reporting system. Sanofi Pasteur provided the human rabies vaccines, financial resources and scientific support for the study. GARC coordinated the overall project from intervention to follow-up and data analysis and worked with the LGU to implement the educational intervention.

### Inclusion

The target population was children enrolled in all 27 public elementary schools of the municipality. The study was launched in June 2011 coinciding with the opening of the school year and active surveillance was concluded by December 2012, with a post-intervention assessment of rabies knowledge conducted in March 2013. The population included in the intervention was the cohort of elementary school children enrolled in Grades 1–5 during the 2011–12 school year. A written informed consent form (ICF) was taken from one parent or legal representative of each child included in the study. Written assent form was gathered from children ≥8 years old, in addition to the parent or legal representative ICF. The protocol of the study has been evaluated and approved by the Ospital ng Palawan Executive Committee.

### Exclusion

The exclusion criteria were children in the last elementary grade, Grade 6, at inclusion time as they would not have been in school during the whole intervention time as specified in the study protocol.

There were a few 15- to 20-year old students who were enrolled in elementary grade levels. Since the target group of the study was specifically children, students aged 15 years and above were excluded from the study, thus only students aged 5 to 14 years at the time of enrolment in the study were included. This allowed us to compare the dog bite incidence between students included in the cohort and patients of similar age group consulting at the ABTC.

### Active surveillance

At enrollment, a questionnaire was completed in order to collect the history of potential rabies exposures (animal bites and scratches) over the past 6 months in the cohort children. Information about a child’s possible rabies exposures was collected retrospectively through home visits, mobile phone contact, and through consultations with midwives and village health workers, teachers and neighbors. During the 18 months of follow-up beginning July 2011, active detection of rabies exposures was conducted every three months until December 2012 and included the interview of the exposed patient’s parent regarding incidents of contact with a suspected rabid animal. Parents were contacted and asked whether their child had experienced contact with a suspect rabid animal since the previous interview and follow-up forms were completed. All patients that had experienced a potential exposure during the previous three months were visited at home and standardized rabies exposure forms (REF) were completed. All follow-up questionnaires administered during the study were similar except the questionnaire provided in March 2012, in which questions regarding the PrEP were added. Also in 2012, a small survey was carried out of the parents of 328 randomly chosen children who were not vaccinated, to determine the reasons for this.

During the follow-up period, the ABTC data were collected on all children with a reported contact with a suspect rabid animal. Every three months thereafter, an ABTC data retrieval form was completed for every rabies exposure consultation for a child included in the study cohort.

### Passive surveillance

Data on animal bite consultations of children in the same age group as the study cohort at the ABTC were collected retrospectively for the period from January 2011 to December 2012 in order to establish a temporal trend of exposures. Bite incidence was calculated from the number of bites amongst children divided by the total number of children aged 5–14 years old based on the 2007 Population Census.

### Interventions

The interventions consisted of complete PrEP vaccination against rabies organized by local health care authorities and provision of education in school on rabies prevention and responsible pet ownership.

### Pre-exposure prophylaxis

The PrEP of the children was organized by the Department of Health and was conducted in January to February 2012. All school children, regardless of whether they were included in the study or not, were offered three intradermal doses of purified Verocell rabies vaccine (VeroRab, Sanofi Pasteur) following the WHO standard PrEP schedule of one dose administered on each of days 0, 7 and 28. The vaccination series was administered in the school premises by the municipal health officer and/or nurses who were trained by DOH. A list of these children given PrEP was maintained at the Provincial Health Office for reference in case of a future bite incident.

### Education intervention

The rabies education intervention entailed the adaptation of the rabies curriculum teachers’ manual that was formalized through the passing of a resolution by the Municipal Mayor. This resolution allowed municipal funds and staff to be used in implementing the integration of rabies and responsible pet ownership in the elementary school curriculum. Actual implementation was achieved by the teachers and local officials of the Department of Education.

Training and orientation sessions regarding the study and processes were conducted for the district superintendent, school nurse, head teachers and Rural Health Unit (RHU) midwives. A Teachers Planning Workshop was conducted for the 27 head teachers from each of the public elementary schools in the municipality, with the objective of developing the implementation plan and strategies to institutionalize the rabies curriculum integration. This was patterned after the successful adaptation of the national prototype by the Bohol provincial rabies education program [9]. Consultative meetings and intense planning sessions with a core group of teachers ensured their continuous involvement and participation. These sessions were conducted to develop new or modify existing entries in the lesson plans thus integrating the local rabies situation in the curriculum manual. Like the Bohol curriculum manual, selected senior teachers were tasked to draft the manual for El Nido. Actual data on rabies cases and animal bite situation in Palawan were used. The draft manual was pre-tested among the other elementary school teachers. The pre-test dealt on the content of the manual as well as the manual’s practical use for the teachers. After two rounds of editing, final copies of the manual were distributed.

The manual was used during the 2012 to 13 school year. It contains modules on rabies which teachers could integrate into Science and Health, Makabayan (Civics, Social Studies, Geography, and History), Filipino, English and Mathematics subjects for the different grade levels throughout the year. An assessment of the impact of the education program was conducted among students in 12 randomly selected elementary schools in the municipality. Pre- and post-intervention tests were conducted among a sample of the students to assess the impact of the education materials, testing the students’ knowledge of rabies, its transmission, dog bite prevention, dog bite management, and responsible pet ownership. The pre-tests were conducted in July 2012 while the post-tests were conducted in March 2013.

**Fig 2:**
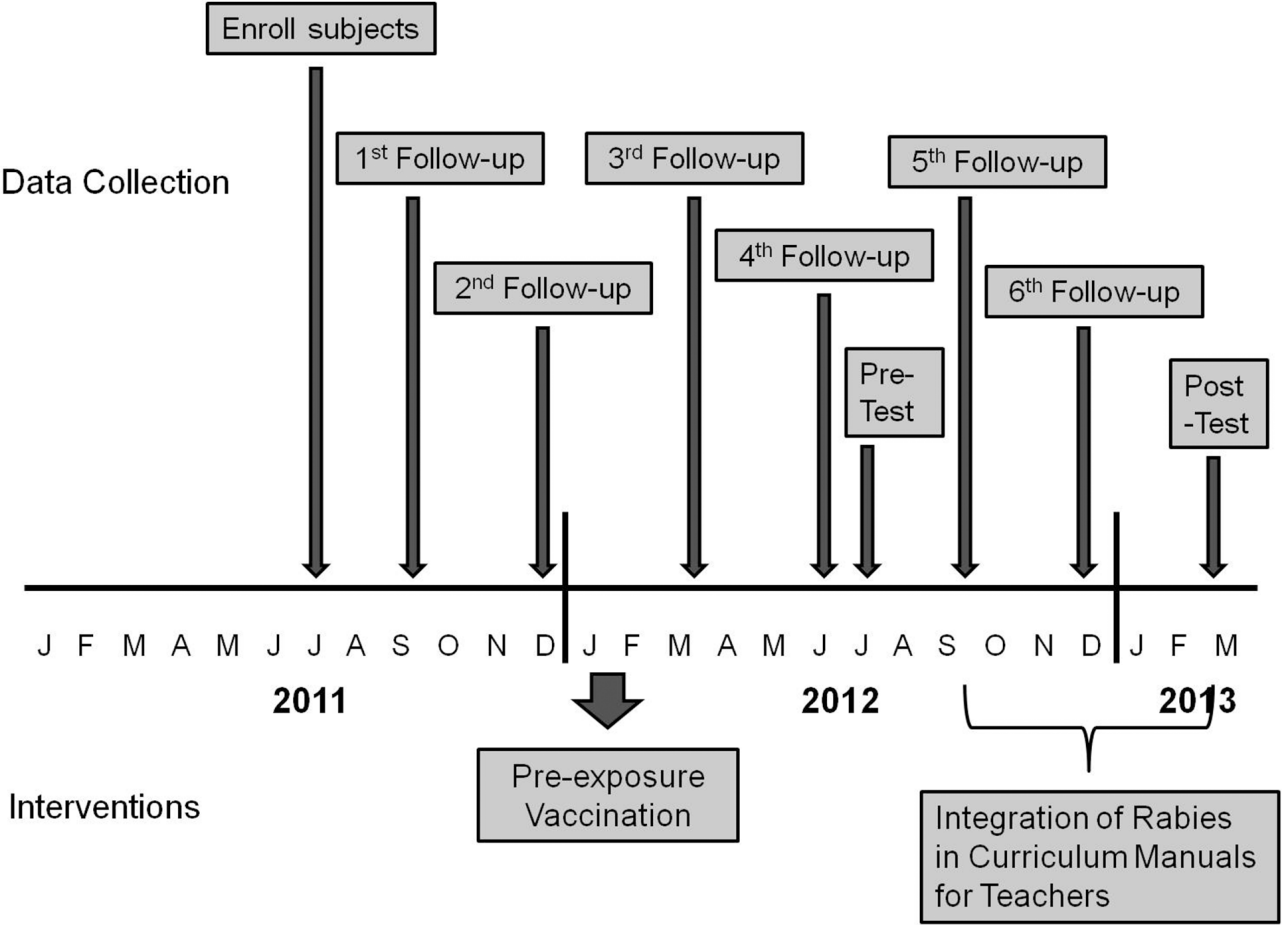
Timeline of study activities.

Fig 2 illustrates the study timeline and various activities undertaken.

### Data analysis

All data was anonymized prior to analysis and Excel was used to generate descriptive statistics, to complete Chi-squared tests on the proportion data, and to perform Mann Whitney U tests on non-parametric data.

### Economic analysis

Costs of PrEP were assessed for the cohort of 4,666 children in the study at the time of PreP administration. PrEP schedule followed the WHO guidelines, with intradermal administration of 0.1 ml volume per site (one site each day) given on days 0, 7 and 28.

The direct medical costs included cost of biologicals (rabies vaccine), cost per shipment, considered as 2% of the vaccine cost, cost of needles, alcohol and cotton used. Costs related to health care staff were not included. Table 1 presents the parameters used for the vaccination cost estimation. The number of doses per vial assumed for the analysis is 4, although in theory 1 vial could be used to deliver 5 x 0.1 mL doses, thus 20% vaccine wastage is built into the calculations.

**Table 1:** Parameters used to estimate cost of PrEP for the study cohort.

Parameters and Costs for PrEP estimation		Values
Parameters	Number of doses required for PrEP	3
	Number of schools	27
	Number of doses per vial	4
Costs (PhP)	Vial (DOH price)	PhP 410
	Dose	PhP 103
	Shipment cost (% of vaccination costs)	2%
	Needle / syringe	PhP 5
	Alcohol (bottle)	PhP 40
	Cotton pack (150 pcs)	PhP 46
	Conversion PhP—USD	PhP 43 –USD 1

In addition, to explore further the benefits of such vaccination program, projected costs of two hypothetical cohorts of children bitten by a dog were assessed according to two scenarios:

(i) Scenario 1, no PrEP:

Assessment of the costs related to the expected annual number of children among the study cohort bitten by a dog, assuming they did not have PrEP. These children were considered to follow the standard of care for Post-Exposure Prophylaxis (PEP) defined as 2-site intradermal method (2-2-2-0-1) for use with PVRV (complete PEP), with RIG given for category III bites.

(ii) Scenario 2, with PrEP

Assessment of the costs related to the expected annual number of children among the study cohort bitten by a dog benefiting from PrEP. Following the national guidelines, such patients should be given only two booster doses on days 0 and 3, but no RIG.

Medical costs for PEP are presented in Table 2. Costs associated with wound care (e.g. antibiotics, tetanus immunization) were not included in the analysis since these do not depend on rabies vaccination status of the patient. Indirect costs were also not included in the analysis.

**Table 2:** Parameters used to estimate costs of PEP according to PrEP status.

Parameters and Costs for PEP estimation		Values
***Full PEP (without PrEP)***	Number of doses required (2-2-2-0-1)	7
	Number of visits	4
	Equine RIG (ERIG) required	2
	% of patients receiving ERIG	15%
	% of patients receiving 7 doses	100%
***Booster PEP (with PrEP)***	Number of doses required (1–1)	2
	Number of visits	2
	ERIG required	0
	% of patients having 2 doses	100%
***PEP Costs***	Vial	PhP 1500
	Number of doses per vial	4
	Dose	PhP 375
	ERIG	PhP 1000
	Alcohol for 1 patient	PhP 10
	Cotton for 1 patient	PhP 2

Time horizon for the comparison of the economic benefit of the two strategies was projected to 1 year, 5 years and 10 years. All medical costs were expressed in terms of Philippine peso (PhP), and converted to US dollars (USD) using the average exchange rate of USD 1 = PhP 43. Table 2 summarizes the parameters used for this analysis.

## Results

### Characteristics of active surveillance study cohort

The total population of 5 to 14 year old children in El Nido according to the National Statistics Office was 9,211. Department of Education records show that 5,764 children (63%) were enrolled in Grades 1 to 5 in the 27 public elementary schools during the 2011 to 12 school year. After the exclusion of 38 students over the age of 15, eighty-two percent (4,700/5,764) of all enrolled children participated in the active surveillance study, with 70% at rural schools and 30% at urban schools. Compared to total school enrollment data, participation rates in the study were significantly higher among students from schools in rural areas (87%) than urban areas (73%), chi-sq test p<0.01. Of the 4,700 students included in July 2011, only 166 (3.5%) dropped out or were lost to follow-up by the end of the study.

At the time of enrolment, study participants were aged 5 to 14 years (median 8.8 years). Just over half, 51% (2,410/4,700) were male. The majority, 65% (3,073/4,700), of students’ households had pet dogs. Among households with dogs, the number of dogs owned ranged from one to 12 dogs (median 2). The majority, 84% (3,965/4,700), of the students’ parents knew of the existence of an ABTC. Of those who knew of the location of the ABTC, 27% lived less than one hour travel time to the ABTC, and the majority (55%) resided within 1–4 hours travel time. (Table 3)

**Table 3:** Demographic characteristics of study cohort (N = 4700).

Characteristics		Number	Percent
**Age group (years)**	5–9	3665	78%
	10–14	1035	22%
**Sex**	Male	2410	51%
	Female	2290	49%
**School location**	Rural	3294	70%
	Urban	1406	30%
**Number of animals in the household**	0	1627	35%
	1	1304	28%
	2 or 3	1436	31%
	4 to 6	301	6%
	7 or more	32	1%
**Travel time from residence to ABTC**	less than one hour	1,253	27%
	one to four hours	2,588	55%
	> 4 hours but < 1 day	4	0.1%
	Unknown	855	18%

### Interventions

#### Pre-exposure prophylaxis (PrEP)

A total of 3,892 elementary school children (82.8% of study cohort and 67% of all children enrolled in school) received a complete (three doses) anti-rabies pre-exposure prophylaxis between January and February 2012. A further 477 children (10.1% of study cohort and 8.3% of all enrollees) received one or two doses and 331 children (7% of the study cohort) received no PrEP (Table 4). School specific complete PrEP coverage rates ranged from 63.5% to 95.3%.

**Table 4:** Number of pre-exposure vaccination doses received by students in El Nido elementary schools based on municipal health office records, January to February 2012.

Number of Doses	Number (percent of study cohort given PrEP) (n = 4,700)	Percentage of all enrolled school children (n = 5,764)
0	331 (7.0%)	5.7%
1	403 (8.6%)	7.0%
2	74 (1.6%)	1.3%
3	3892 (82.8%)	67.5%

A survey of 328 children who did not get vaccination revealed that the major reason for non-vaccination of children in the study cohort was absence during scheduled vaccination day (applying to 69% of those surveyed, Table 5).

**Table 5:** Reasons for non-vaccination of children (N = 328) in school during mass vaccination campaign in El Nido elementary schools, January and February 2012.

Reason	Number (%) of students
Absence in school at time of vaccination	227 (69%)
Already vaccinated prior to school-based intervention	75 (23%)
Transferred to another school	18 (6%)
Parents refused to have child vaccinated	4 (1%)
Other reasons (school drop-out, unknown)	4 (1%)
TOTAL	328 (100%)

#### School-based rabies education

Almost 100% (4,663/4,666) of parents surveyed in March 2012 reported that their child had received information about rabies in their school. One parent did not know whether their child had received rabies education in school. Information was missing from two of the respondents.

A total of 255 students from 12 randomly selected elementary schools, answered the pre-test while 293 students answered the post-test. Fifty-seven percent (145/255) of those who answered the pre-test were male while 52% (159/293) of those who answered the post-test were male (no sign. difference, chi sq, 1df, p>0.05).

**Fig 3:**
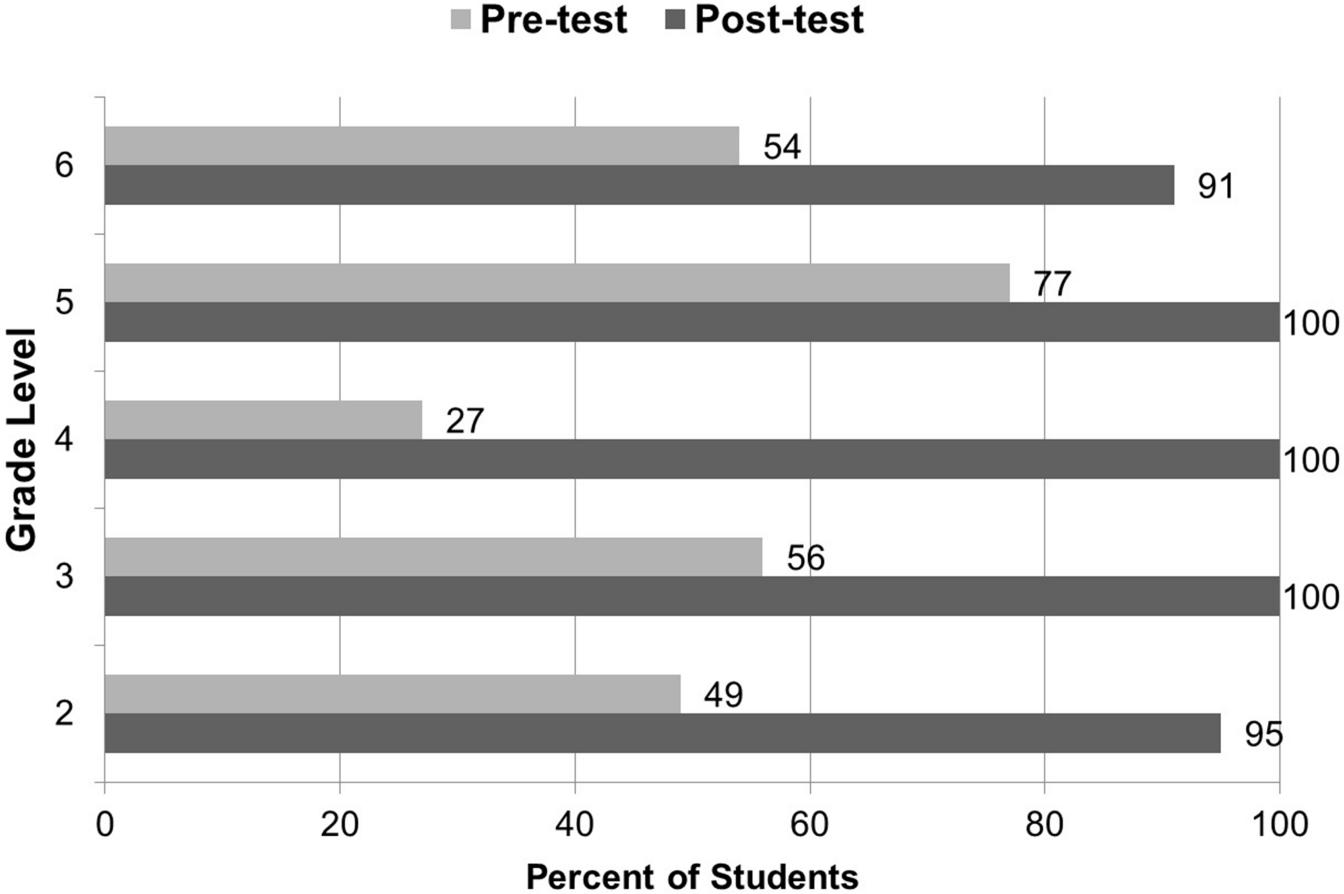
Percentages of students who knew about rabies based on pre and post-tests of students in El Nido elementary schools, school year 2012–13.

At the end of the school year, there was a significant increase in the proportion of students who were aware of the disease of rabies. In the pre-test, overall 49% (95% CI: 43–55%) of students were aware of rabies while in the post-test 98% (95% CI: 95–99%) reported that they were aware of rabies (chi sq. 1df, p<0.01). At every grade level, there was a significant increase in the proportion of students who were aware of rabies (Fig 3).

**Fig 4:**
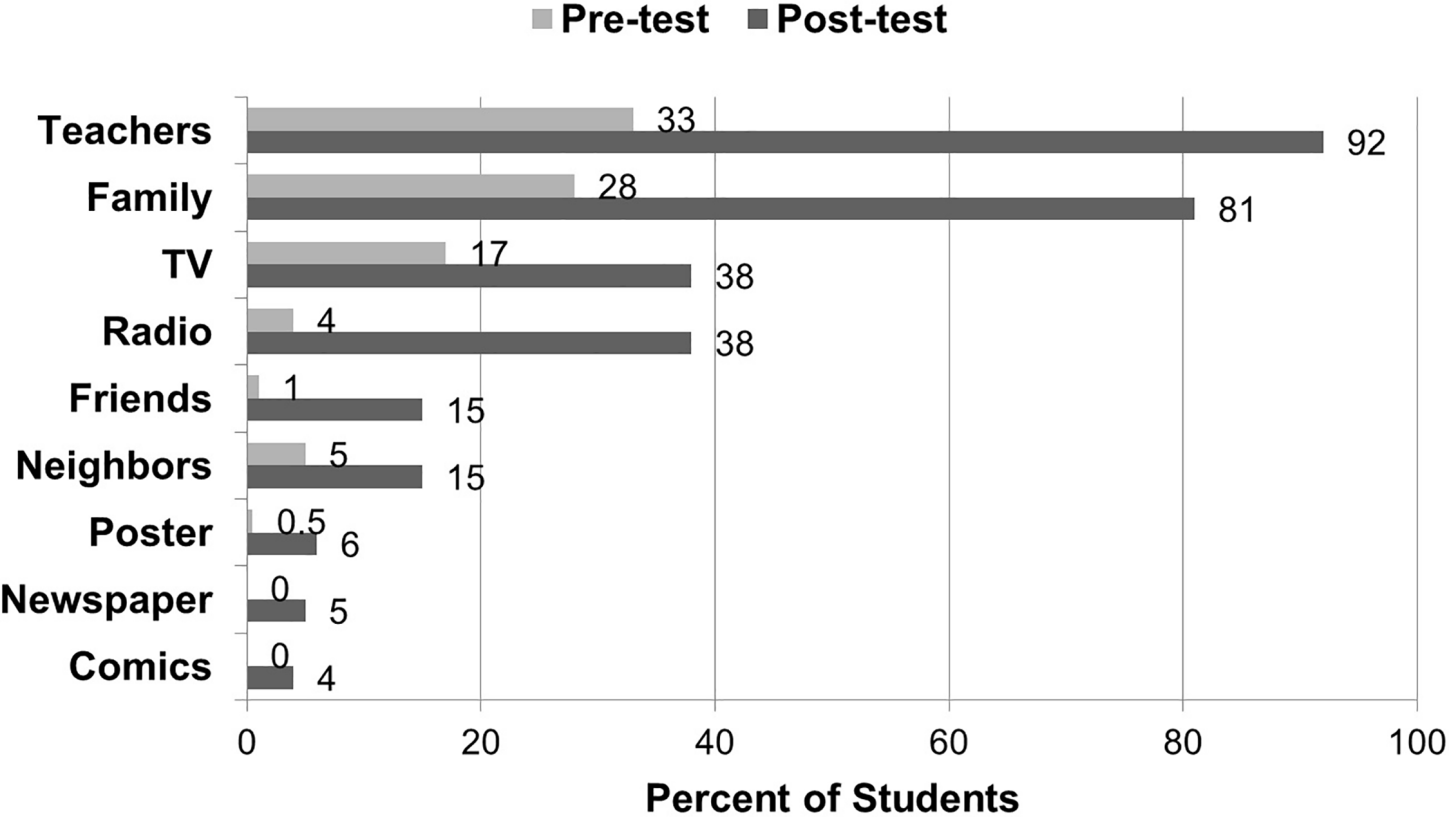
Sources of information on rabies of elementary school students in El Nido, July 2012 and March 2013.

Teachers and family members were the main sources of information about rabies for the students. The proportion of students reporting that teachers were a source of information on rabies increased significantly from 33% (95% CI: 27–39%) during the pre-test to 92% (95% CI: 88–95%) during the post-test (chi sq. 1df, p<0.01, Fig 4).

#### Active surveillance of study cohort

**Fig 5:**
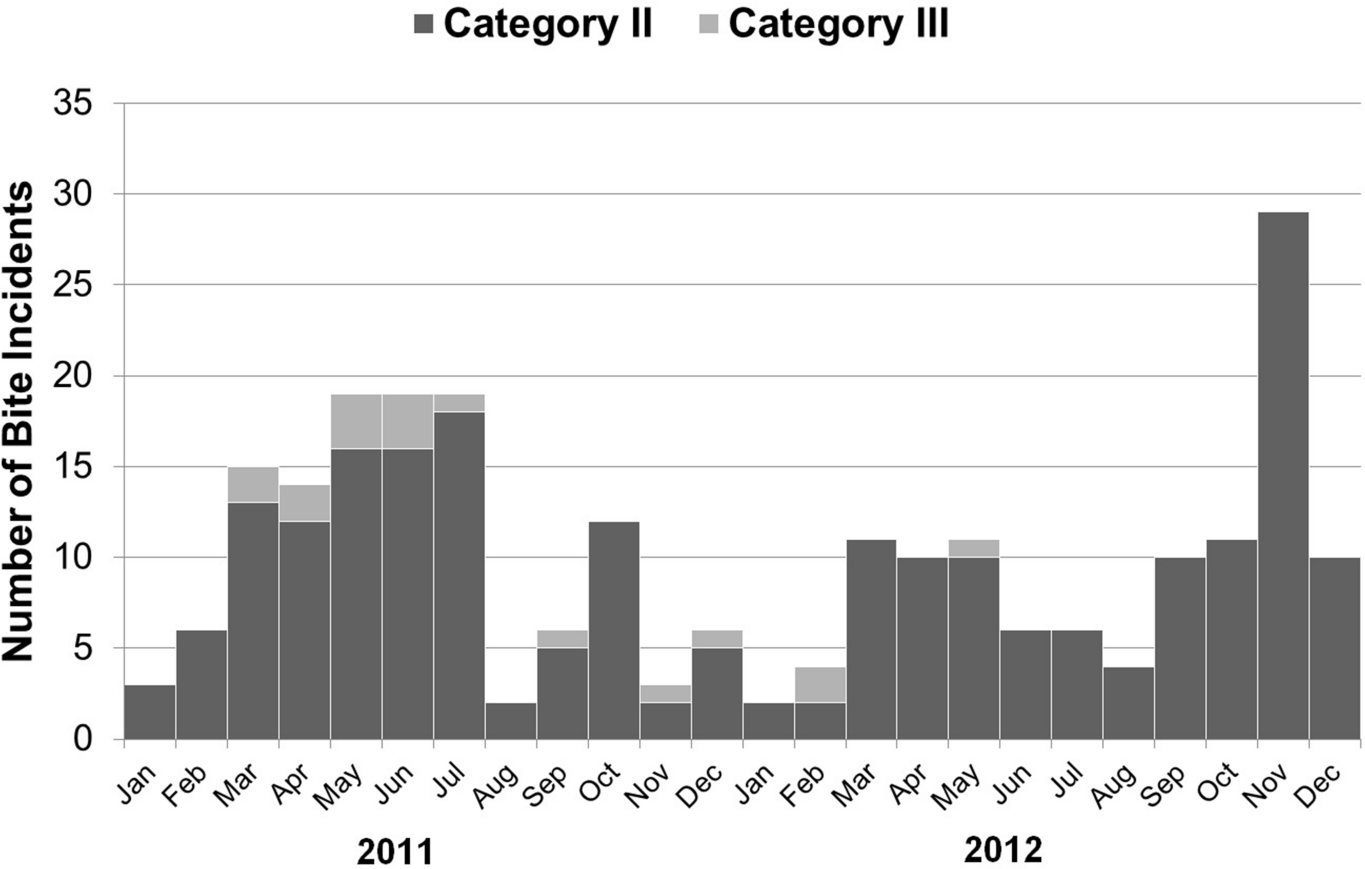
Number and type of bites reported by study cohort, January 2011 to December 2012.

From January 2011 to December 2012, there were 238 potential rabies exposures reported during follow-up interviews, i.e. category II or III bites reported among the study cohort (Fig 5). The majority (93%) of these were category II bites (Table 6) and the majority, 70% (167/238) of the animal bite incidents involved dogs.

**Table 6:** Number and type of animal bites among the study cohort using active surveillance, 2011–2012.

Bite Category	2011	2012	Total
II	110 (89%)	111 (97%)	221 (93%)
III	14 (11%)	3 (3%)	17 (7%)
**Total**	**124 (100%)**	**114 (100%)**	**238 (100%)**

There were slightly fewer bites in 2012 than in 2011 (114, compared to 124). However, an unusual spike in incidents in November 2012 occurred following a typhoon (Fig 5). If this month’s bites had been an average of those in October and December 2012 (11 bites), the total for 2012 would have been only 96 bites, a 22% decline. There was a significant decrease in the proportion of category III bites from 11% in 2011 to 3% in 2012 (chi-sq., 1df p<0.05).

Only 27% (65/238) of animal bite patients from the study cohort were brought to the ABTC. In 2011, this was 40 patients, with a time lapse from bite incident to consultation at the ABTC ranging from 0 to 145 days (mean 13.35, median 2 days). In 2012, this was 25 patients, with a time lapse ranging from 0 to 32 days (mean 5.4, median 3 days). There was no significant difference between the 2011 and 2012 medians using Mann Whitney U Test (p>0.05). There was a significant increase in the proportion of those who had their wounds washed with soap and water (60% in 2011 vs 77% in 2012, chi sq. 1df, p<0.01) and a significant decrease in the proportion of those given other traditional remedies (10% in 2011 vs 2% in 2012. Chi sq. 1df, p<0.01).

Of the 40 cohort patients consulting the ABTC in 2011 (prior to the study PrEP intervention), 39 received the full PEP regimen (TRC Intradermal, 2-site) and only 1 was considered pre-immunized and received the two booster dose PEP regimen. Of the 25 consulting the ABTC in 2012, 5 were not prescribed PEP, 7 received the full PEP regimen (two in January 2012), and 13 were considered pre-immunised as a result of the study and received the two booster dose PEP regimen. Thus of the 18 patients who could have been preimmunised by the intervention, 13 were given the abbreviated two booster PEP regimen. Three patients with Category III bites (2 in 2011 and 2012) also received equine rabies immunoglobulin (ERIG) because they were not pre-immunised. No child was hospitalized after being bitten by an animal and all children survived.

#### Passive surveillance at animal bite treatment center (ABTC)

A review of the records at the ABTC revealed a total of 205 patient consultations for animal bites in 2011. In 2012, there were 212 consultations. Children aged 5–14 years comprised 39% (79) and 33% (69) of the patients in 2011 and 2012, respectively.

The majority (62%) of the children bitten by animals were boys. Most of the bites were Category II bites (Table 7).

**Table 7:** Number of dog bite consultations of 5–14 year old children at the El Nido animal bite treatment center by category using passive surveillance, 2011 to 2012.

Category	2011	2012	Total (Percent)
II	65	44	109 (74%)
III	8	14	22 (15%)
Unspecified	6	11	17 (11%)
**TOTAL**	**79**	**69**	**148 (100%)**

In 2011, the time lapse from bite incident to consultation at the ABTC ranged from 0 to 155 days (mean 10.7 days, median 2 days). In 2012, the time lapse ranged from 0 to 21 days (mean 3.0 days, median 2 days,). Mann Whitney U Test shows no significant difference between the 2011 and 2012 medians (p>0.05).

#### Comparison of active and passive surveillance

**Fig 6:**
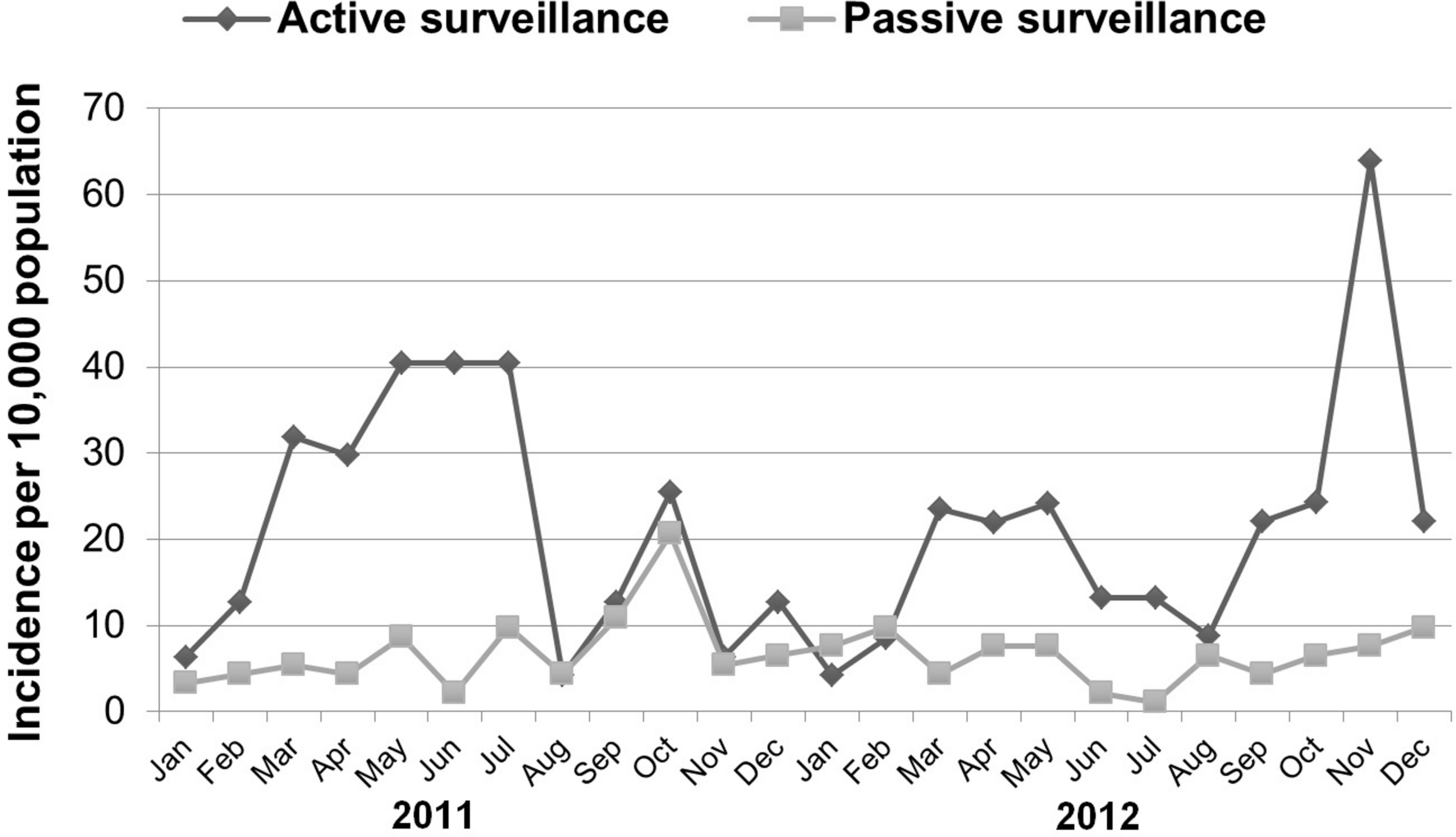
Incidence of category II and III dog bites among 5–14 year old children in El Nido estimated from passive and active surveillance, 2011 to 2012.

The active surveillance cohort size in this study was 4,700, and the total number of similar aged children in the municipality was 9,211. The number of bites detected by active surveillance corresponded to annual bite incidences of 26.4 and 24.7 / 1,000 children for 2011 and 2012 respectively. In contrast, the passive surveillance data from the ABTC recorded 8.6 and 7.5 bite incidents / 1,000 children for 2011 and 2012 respectively (Table 8). Thus, active surveillance consistently revealed a three-fold higher incidence of animal bites among children than that estimated from consultations at the ABTC alone (Table 8, Fig 6, chi sq. 1df, p<0.01).

**Table 8:** Estimated dog bite incidence among 5–14 year old children in El Nido using passive and active surveillance, 2011 and 2012.

Type of surveillance (Data source)	Number and Incidence of Dog Bites		Remarks
	2011	2012	
Passive surveillance (based on ABTC records, n = 9211)	79 incidents (8.6/1,000 children)	69 incidents (7.5/1,000 children)	no significant decrease (p = 0.46)
Active surveillance (based on follow-up of study cohort, n = 4,700)	124 incidents (26.4/1,000 children)	114 incidents (24.7/1,000 children)	no significant decrease (p = 0.65)
Active surveillance incidence as a percentage of passive surveillance incidence	307%	329%	

Both passive and active surveillance revealed a slight decrease in animal bite incidence among 5–14 year old children in 2012 compared to 2011. However, the decrease in incidence was not statistically significant (Table 8).

### Economic impact of PrEP

According to the national animal bite management protocol, persons that have received PrEP require only two booster doses following subsequent exposures and no RIG necessary for Category III bites [1,6]. Thus, there are substantial *potential* cost savings in terms of vaccine and biologicals.

#### Pre-exposure prophylaxis (PrEP) campaign

During the vaccination program, 12,227 doses of anti-rabies PrEP were administered, a total of 3,055 vials. Across the 27 selected schools, 3,892 children received the complete schedule of rabies vaccine (i.e. three doses). The total cost of this vaccination program (excluding salaries) was USD 31,588 (PhP 1,345,653) or $8.11 per fully vaccinated child, of which 93% accounted for vaccine cost (Table 9).

**Table 9:** Vaccination costs related to anti-rabies PrEP among the study cohort.

Costs of PrEP	PhP	USD
Vaccine cost	PHP 1,252,517	$29,402
Shipment cost	PHP 25,050.34	$588
Needle/syringe	PHP 61,098	$1,434
Alcohol	PHP 3,240	$76
Cotton	PHP 3,747	$88
**Total**	**PHP 1,345,653**	**$31,588**

#### Post-exposure prophylaxis (PEP) following PrEP

Assuming an animal bite incidence rate of 2.56% per year, (from our active surveillance results) among the children given complete PrEP, 100 of them would need PEP each year. We assumed that 100% of the exposed children received the full schedule of rabies vaccination, either for the complete PEP or booster PEP situations. Only 15% of exposed children received ERIG in the complete PEP situation, and none under the booster PEP situation.

Under our assumptions, the total cost of regular PEP for a child with no PrEP was PHP 2,860 ($67.12) and PEP boosting for a child who already had PrEP was PHP 784 ($18.40).

When considering a complete course of PEP (i.e. for children not having benefited from PrEP), the cost of care for these 100 children over the first year would be **PHP 285,950 (USD 6,712)**. The cost in the context of booster PEP only (i.e. for children having benefited from PrEP) would be PHP 78,400 (USD1,840) (Table 10).

**Table 10:** Costs of PEP according to PrEP status over 1 year.

	Scenario 1 No PrEP			Scenario 2 With PrEP		
	Number	Costs		Number	Costs	
		PhP	USD		PhP	USD
Number of children bitten	100	-	-	100	-	-
Number of PrEP vaccination (per dose)	0	0	0	12227	PHP 1,345,653	$31,588
Number of PEP vaccine doses	700	PHP 262,500	$6,162	200	PHP 75,000	$1,761
ERIG	15	PHP 15,000	$352	0	PHP 0	$0
Needle / Syringe	730	PHP 3,650	$86	200	PHP 1,000.00	$23
Alcohol	400	PHP 4,000	$94	200	PHP 2,000	$47
Cotton	400	PHP 800	$19	200	PHP 400	$9
***Subtotal (w/o PrEP vaccination costs)***	** **	**PHP 285,950**	**$6,712**	** **	**PHP 78,400**	**$1,840**
**Total (w/ PrEP vaccination costs)**				**-**	**PHP 1,424,053**	**$33,428**

The PrEP scenario incurs the additional costs of PrEP (USD 31,588) for the first year only and the cost of booster only PEP (USD 1,840) every year. The PEP only scenario costs USD 6,712 every year for 100 children exposed (assuming no children are re-exposed). Thus over a ten year time horizon, the PrEP scenario produces a cumulative cost-savings of USD 17,133, compared to 10 years of providing PEP to 100 children every year.

## Discussion / Conclusion

The study found that most children with animal bites do not seek treatment at the ABTC. The bite incidence assessed by active surveillance (26.4 and 24.7 bites /person year for 2011 and 2012 respectively) was around 3 times higher than that assessed by passive surveillance in the ABTCs (8.6 and 7.5 bites/person year for 2011 and 2012), which agrees well with the active surveillance data that only 27% of bites were reported to the ABTC. With only one ATBC in the province, it is expected that the bite incidents reported to ABTCs which were detected in our active surveillance were also recorded in the passive surveillance records. The passive surveillance data would also include those bite incidents amongst the small number of schoolchildren not enrolled in this study, and more importantly the significant proportion of children (37%) not enrolled in schools. It is important to note that unless out of school children are made aware of the risk of acquiring rabies from animal bites and encouraged to report bite incidents, they may fail to inform their parents or guardians about such incidents and consequently not receive treatment.

Dog bites in the study cohort were generally more common from March to August when children are out of school, and also atypically peaked in November 2012 when as a result of a typhoon schools were closed. There was a slight, but not significant decrease in incidence of dog bites between 2011 and 2012 among the study cohort. The increase in bite incidents in November 2012 (due to a typhoon that occurred that month leading to the displacement of some dogs and increasing the probability of children encountering stray dogs) masked an underlying decrease in bite incidents in the study cohort between 2011 and 2012. The significant decrease in category III bites among the study cohort may indicate that students were beginning to practice some of the avoidance behaviors taught in school, e.g. not approaching or moving away from aggressive animals. There was no active veterinary intervention (dog vaccination) therefore it is unlikely that there was a change in the underlying epizootiology of animal rabies in the area.

The community sources for surveillance data were the midwives and village health workers who are often good primary source of health information because most of them live in the communities that they serve and are well known to the community. They were able to collect information early and relayed these data to the ABTC. Teachers often knew of bite incidents among their students, and most teachers reported the bites to the village health worker or the ABTC because of their improved awareness and realization of the need for urgent medical attention.

PrEP is a life-saving intervention that could save the lives of children living at constant risk of exposure to rabies in enzootic regions. This early intervention brings protection in case of unapparent or unreported exposure, in case of delay in post-exposure therapy or in case of non-availability of RIG. Thus, although booster doses are recommended in the event of an exposure, it is possible that children that have received PrEP are less likely to die of a rabies exposure, in case of delayed or even a lack of access to PEP [10].

Besides providing immune protection, preventive rabies vaccination has a clear potential advantage in terms of cost effectiveness in populations at increased risk of rabies exposure. One previous study has reported that by vaccinating children with PrEP, there are significant future cost savings [11].

Here we estimated potential cost saving of USD 17,133 over 10 years. However, the realisation of such savings depends critically on two things. First the cohort children bitten would need to present at the ABTC for every bite (which they did not in this study). Second, the ABTCs need to the access and use records of PrEP vaccination to administer the reduced PEP booster regimen where appropriate. There is evidence that the record keeping was adequate, and that these cost savings were beginning to be realized. A total of 13 bite victims (out of a possible 18) that were part of the cohort offered PrEP were only given a 2- dose booster regimen when they were bitten in 2012. PrEP could be a short term cost saving strategy for many remote rabies endemic areas where access to an ABTC is difficult and dog vaccination efforts are far from reaching elimination of the disease.

There are several limitations to this study. There could have been unrecalled bite incidents by both parent and child, or a child could have failed to report a bite. Integrating rabies information in the curriculum requires supplemental teaching materials and additional time for follow-up to measure impact. The school-based intervention failed to reach the population of children that were not enrolled in a formal educational system and may be at higher than average risk of exposure to rabies. Thus our active surveillance (of school children) is not completely comparable to the passive surveillance (which includes all children aged 5–14). However, compared to school children, those out of school may suffer even higher bite incidence rates, and lower rates of presentation at the ABTC. The government rabies control and prevention program should consider expanding the strategy of providing PrEP for at-risk populations including children who are out of school.

This study demonstrated the clear advantage of PrEP in selected population groups, i.e. school-aged children in rabies endemic areas with limited access to animal and human rabies prevention and control services. A longer follow-up of the cohort is recommended to assess the longer term impact of the preventive vaccination and education interventions. Rabies prevention education for children who are not enrolled in formal educational programs should not be neglected and should be included in community education strategies.

Rabies is a vaccine-preventable disease in humans and animals. The tools to implement strategies to successfully control rabies are available [12,13]. The strengthening and expansion of veterinary interventions to target the disease at source cannot be overemphasized. In enzootic countries, the human rabies vaccination is usually administered only after exposure to rabies suspected animal, when for the most exposed and sensitive populations an earlier intervention can be proposed through the implementation of preventive vaccination.

## References

[1] WHO Expert Consultation on rabies: Second report (2013). Geneva. 1–88 p. Available: http://www.ncbi.nlm.nih.gov/pubmed/24069724. 24069724

[2] Miranda ME, Meslin FX (2006) Human rabies prevention. Dev Biol 125: 155–164. Available: http://www.ncbi.nlm.nih.gov/pubmed/16878473. 16878473

[3] Knobel DL, Cleaveland S, Coleman PG, Fevre EM, Meltzer MI, et al (2005) Re-evaluating the burden of rabies in Africa and Asia. Bull World Heal Organ 83: 360–368. Available: http://www.ncbi.nlm.nih.gov/pubmed/15976877. PMC2626230 15976877

[4] Department of Health Philippines (2012) National Rabies Prevention and Control Program: Manual of Operations. Available: http://www.doh.gov.ph/sites/default/files/FINALMOP6.4.13WORDRADMay30.pdf.

[5] Philippines G of the (2007) Anti-Rabies Act of 2007: An Act Providing for the Control and Elimination of Human and Animal Rabies. Republic A. Available: http://www.senate.gov.ph/lis/pdf_sys.aspx?congress=13&type=republic_act&p=1.

[6] Department of Health Philippines (2009) AO No.2009-0027 Amendment to Revised Guidelines on ABM.pdf. Available: http://rabiespoi.org/index.php?option=com_content&view=article&id=51&Itemid=43.

[7] World Health Organization (2005) WHO Expert Consultation on Rabies: First Report. Available: http://www.who.int/rabies/trs931_06_05.pdf. 16485446

[8] Chulasugandha P, Khawplod P, Havanond P, Wilde H (2006) Cost comparison of rabies pre-exposure vaccination with post-exposure treatment in Thai children. Vaccine 24: 1478–1482. doi: 10.1016/j.vaccine.2005.03.059 16221511 10.1016/j.vaccine.2005.03.059

[9] Lapiz SM, Miranda ME, Garcia RG, Daguro LI, Paman MD, et al (2012) Implementation of an intersectoral program to eliminate human and canine rabies: the Bohol Rabies Prevention and Elimination Project. PLoS Negl Trop Dis 6: e1891 Available: http://www.ncbi.nlm.nih.gov/pubmed/23236525. doi: 10.1371/journal.pntd.0001891 23236525 10.1371/journal.pntd.0001891 PMC3516573

[10] Kessels JA, Recuenco S, Navarro-Vela AM, Deray R, Vigilato M, et al (2017) Pre-exposure rabies prophylaxis: a systematic review. Bull World Health Organ 95: 210–219C. http://dx.doi.org/10.2471/BLT.16.173039. 28250534 10.2471/BLT.16.173039 PMC5328107

[11] Lang J, Plotkin S (1997) Rabies risk and immunoprophylaxis in children. Adv Pediatr Infect Dis: 219–255. 9544314

[12] Strategies for the control and elimination of rabies in Asia: Report of a WHO Interregional Consultation (2001). Geneva. Available: http://www.who.int/rabies/en/Strategies_for_the_control_and_elimination_of_rabies_in_Asia.pdf.

[13] Rabies vaccines: WHO position paper (2010). Wkly Epidemiol Rec 85: 309–320. Available: http://www.who.int/wer/2010/wer8532.pdf?ua=1.

